# Simple Hyperintensional Belief Revision

**DOI:** 10.1007/s10670-018-9971-1

**Published:** 2018-02-05

**Authors:** F. Berto

**Affiliations:** Department of Philosophy, Institute for Logic, Language and Computation (ILLC), University of Amsterdam, Oude Turfmarkt 141-147, 1012 GC Amsterdam, The Netherlands

**Keywords:** Framing effects, Hyperintensionality, Belief revision, Non-monotonic reasoning, Doxastic logic, Epistemic logic, Inconsistent belief management

## Abstract

I present a possible worlds semantics for a hyperintensional belief revision operator, which reduces the logical idealization of cognitive agents affecting similar operators in doxastic and epistemic logics, as well as in standard AGM belief revision theory. (Revised) belief states are not closed under classical logical consequence; revising by inconsistent information does not perforce lead to trivialization; and revision can be subject to ‘framing effects’: logically or necessarily equivalent contents can lead to different revisions. Such results are obtained without resorting to non-classical logics, or to non-normal or impossible worlds semantics. The framework combines, instead, a standard semantics for propositional S5 with a simple mereology of contents.

## Introduction

Standard AGM belief revision theory imposes a high amount of idealization on cognitive agents who revise their beliefs in the light of new information^1^. The first postulate for belief revision in Alchourrón et al. (1985), (${{\hbox {K}}}*1$), has it that $K * \phi$ (belief set *K* after revision by $\phi$) is closed under the full strength of classical logical consequence. Postulate (${{\hbox {K}}}*5$) trivializes belief sets revised in the light of inconsistent information^2^: if $\phi$ is a logical inconsistency, then $K * \phi = K_{\bot }$, the trivial belief set; agents who revise via inconsistent inputs trivially believe everything. And postulate (${{\hbox {K}}}*6$) requires that, if $\phi$ and $\psi$ are logically equivalent, then $K * \phi = K * \psi$, that is, revising by either gives the same belief set.

These principles are rather implausible for agents like us all. Against (${{\hbox {K}}}*1$), our belief states need not be closed under classical logical consequence (perhaps under any kind of monotonic logical consequence: see e.g. Jago (2014) for extended discussion). Against (${{\hbox {K}}}*5$), we do not trivially believe everything just because we occasionally hold inconsistent beliefs, and we should not be modeled as undergoing a trivialization of our belief system just because we can be, as we occasionally are, exposed to inconsistent information. Against (${{\hbox {K}}}*6$), it is well known that how we revise our beliefs, as well as our preferences, is subject to what psychologists call *framing effects* (Kahneman and Tversky 1984): logically or necessarily equivalent contents can trigger different revisions depending on how they are presented. Agents may revise their beliefs in one way when told they have 60% chances of succeeding in a task, in another way when told they have 40% chances of failing.

There are obvious connections between such idealization phenomena and the so-called problem of *logical omniscience*: a cluster of closure conditions on knowledge and/or belief, which come as a spin-off of the characterization of such notions as restricted quantifiers over possible worlds employed since Hintikka (1962):

(H) $B \phi$ is true at *w* iff $\phi$ is true at all $w_{1}$ such that $wRw_{1}$

Here *B* stands for the relevant (non-agent-indexed) cognitive state (knowledge, belief); $R \subseteq W \times W$ is the usual accessibility relation on the space of worlds *W*. Cognitive agents know or believe all logical truths, know or believe all the logical consequences of what they know or believe, and never have inconsistent beliefs. It is widely agreed (see e.g. Fagin et al. 1995, pp. 34–35; Meyer 2001, p. 186) that this gives implausibly idealized notions of knowledge and belief, having little to do with human cognition.^3^

While work on belief *bases* (sets of beliefs not closed under logical consequence, see Hansson 1999) has gone a long way towards reducing the idealization in the original AGM approach, this is still very much present in static and dynamic doxastic and epistemic logics, which aim at recapturing AGM within a modal language with appropriate formal semantics and proof theory. The reason is, plainly, that such approaches build their conditional belief or dynamic belief revision operators on top of a largely standard, normal modal framework.

Works such as Spohn (1988), Segerberg (1995), Lindström and Rabinowicz (1999), Board (2004), van Ditmarsch (2005), Asheim and Sövik (2005), Leitgeb and Segerberg (2005), van Benthem (2007, 2011), van Ditmarsch et al. (2007), Baltag and Smets (2008), Girard and Rott (2014), usually include operators for conditional belief, $B^{\phi }\psi$ (‘Conditional on $\phi$, it is believed that $\psi$’, or ‘It is believed that $\psi$ after receiving the information that $\phi$’), or for belief revision, $[*\phi ]B\psi$ (‘After revision by $\phi$, it is believed that $\psi$’), which closely mirror the original AGM postulates. As for (${{\hbox {K}}}*6$), for instance, such logics will typically satisfy (model-theoretic) principles like the following:

- If $$\vDash \phi \leftrightarrow \psi $$, then $$\vDash [* \phi ]\chi \leftrightarrow [* \psi ]\chi $$
- If $$\vDash \phi \leftrightarrow \psi $$, then $$\vDash B^{\phi }\chi \leftrightarrow B^{\psi }\chi $$

Such modal operators for belief revision or conditional belief are, thus, *merely* intensional, that is, incapable of detecting differences (often called *hyperintensional*) more fine-grained than ordinary modal ones: when $\phi$ and $\psi$ are logically or necessarily equivalent, they can be replaced *salva veritate* as indexes in $[*\ldots ]$ and $B^{\ldots }$. However, again, this is not how real people revise their beliefs and plausibility judgments.

The framing effect is but a special case of a broader fact: thought is hyperintensional. Our (propositional) mental states—believing, supposing, desiring, hoping, fearing—can treat logically or necessarily equivalent contents differently: Lois Lane can wish that Superman is in love with her without wishing that Clark Kent is in love with her, although (if Barcan Marcus and Kripke are right) it is metaphysically impossible for Superman to be other than Clark Kent. We can conceive that $75 \times 12 = 900$ without conceiving that Fermat’s Last Theorem is true; but given the necessity of mathematical truths, the two make for the same content or proposition in possible worlds semantics: the total set of worlds.

In *this* paper, I aim at modeling agents whose belief revision processes are sensitive to framing effects; who can hold inconsistent beliefs without thereby believing everything; who are safe from various other forms of logical omniscience.^4^ Conservative logicians may take as an advantage of the approach proposed below, that it models the aforementioned hyperintensional phenomena without weakening the underlying classical and (normal) modal logic (as it happens, e.g., in *paraconsistent* logics for the management of inconsistent information: see Tanaka et al. 2013), and without resorting to so-called non-normal or impossible worlds (Kiourti 2010; Berto 2013), which have often been proposed as a tool to deal with logical omniscience (see Rantala 1982; Wansing 1990; Nolan 1997; Jago 2007, 2014). It employs, instead, only semantic notions acceptable by, and already available in the toolkit of, a classical modal logician (I will mention, though, a revenge of non-classical frameworks in a footnote below).

Another feature of the approach is its simplicity. It takes its cue from a single, plain insight: we should take at face value the view of beliefs as (propositional) representational mental states bearing *intentionality*, that is, being *about* states of affairs, situations, or circumstances which make for their content. Arguably, it is precisely the aboutness of such intentional states that can account for many of their hyperintensional features: when we think that $75 \times 12 = 900$, our thought is about these very integers, not about diophantine equations, elliptical curves, or else. As we will see, the discriminating powers of the standard possible worlds apparatus may improve when we supplement it with a simple mereology of contents—what the relevant intentional (doxastic) states are about.

The semantics proposed below makes for a very basic set-up: we consider a single-agent setting with a propositional language and only one binary operator expressing conditional belief, or disposition to belief revision, $B^{\phi }\psi$ [read: ‘Conditional on $\phi$, the agent believes $\psi$’, or: ‘After revising by $\phi$, the agent believes $\psi$’: belief revision is understood as ‘belief change in the indicative (conditional) mood’, Leitgeb and Segerberg 2005, p. 186]. In this, we follow the framework of non-dynamic doxastic logics such as Board (2004) (see also Asheim and Sövik 2005; Bonanno 2005). *B* is understood, as we will see, as a variably strict quantifier over possible worlds with a content-containment requirement. It involves no model transformations, as it happens in dynamic epistemic logics such as van Benthem (2007) and Baltag and Smets (2008) (for an overview, see van Benthem and Smets 2015). The latter approach has the advantage of embedding information on how the plausibility agents assign to scenarios is globally re-arranged after belief revision, thus allowing the modeling of iterated revisions (see the discussion in van Ditmarsch 2005, pp. 269–270). But the former approach, à la Board, has the advantage of allowing a simple inspection of the hyperintensional features of our operator. How to expand our semantics in various dynamic directions may be the subject of future work.

## A Hyperintensional Semantics

We have a propositional language ${\mathcal {L}}$ with an indefinitely large set ${\mathcal {L}}_{AT}$ of atomic formulas, *p*, *q*, *r* ($p_{1}, p_{2}, \ldots$), negation $\lnot$, conjunction $\wedge$, disjunction $\vee$, a strict conditional $\prec$, the belief operator *B*, round parentheses as auxiliary symbols (, ). We use $\phi$, $\psi$, $\chi$,..., as metavariables for formulas of ${\mathcal {L}}$. The well-formed formulas are items in ${\mathcal {L}}_{AT}$ and, if $\phi$ and $\psi$ are formulas:

$$\begin{aligned} \lnot \phi \ | \ (\phi \wedge \psi ) \ | \ (\phi \vee \psi ) \ | \ (\phi \prec \psi ) \ | \ B^{\phi }\psi \end{aligned}$$

We identify ${\mathcal {L}}$ with the set of its well-formed formulas. A *frame* for ${\mathcal {L}}$ is a tuple ${\mathfrak {F}}$ = $\langle W, \{R_{\phi } \ | \ \phi \in {\mathcal {L}}\}, {\mathcal {C}}, \oplus , c \rangle$, understood as follows. *W* is a set of possible worlds. $\{R_{\phi } \ | \ \phi \in {\mathcal {L}}\}$ is a set of accessibilities between worlds, where each $\phi \in {\mathcal {L}}$ has its own $R_{\phi } \subseteq W \times W$. These satisfy a number of conditions, to which we come in a minute. ${\mathcal {C}}$ is a set of *contents*. We should understand contents as the situations (the configurations of objects and properties) the formulas of ${\mathcal {L}}$ involved in belief ascriptions are *about*. In the metalanguage I use variables $w, w_{1}, w_{2}, \ldots$, ranging over possible worlds, $x, y, z \ (x_{1}, x_{2}, \ldots )$, ranging over contents, as well as the symbols $\Rightarrow , \Leftrightarrow , \& , or, \sim , \forall , \exists$, with the usual reading. $\oplus$ is *content fusion*, a binary operation on ${\mathcal {C}}$ making of contents part of larger contents and satisfying, for all $xyz \in {\mathcal {C}}$:

- (Idempotence) $$x \oplus x = x$$
- (Commutativity) $$x \oplus y = y \oplus x$$
- (Associativity) $$(x \oplus y) \oplus z = x \oplus (y \oplus z)$$

We assume unrestricted fusion, that is, $\oplus$ is always defined on ${\mathcal {C}}$: $\forall xy \in {\mathcal {C}} \ \exists z \in {\mathcal {C}} (z = x \oplus y)$. We then define *content parthood*, $\le$, the usual way: $\forall xy \in {\mathcal {C}} (x \le y \Leftrightarrow x \oplus y = y)$. This makes of parthood a partial ordering—for all $xyz \in {\mathcal {C}}$:

- (Reflexivity) $$x \le x$$
- (Antisymmetry) $$ x \le y \ \& \ y \le x \Rightarrow x = y$$
- (Transitivity) $$ x \le y \ \& \ y \le z \Rightarrow x \le z$$

Thus, $\langle {\mathcal {C}}, \oplus \rangle$ is a join semilattice. We may also assume that ${\mathcal {C}}$ is complete: any set of contents $S \subseteq {\mathcal {C}}$ has a fusion $\oplus S$. Finally, we can think of all contents in ${\mathcal {C}}$ as built via fusions out of *atoms*, contents with no proper parts ($Atom(x) \Leftrightarrow \ \sim \exists y (y < x)$, with < the strict order defined from $\le$), which we stipulate to be at the bottom of our semilattice.

$\langle {\mathcal {C}}, \oplus \rangle$ is needed to assign contents to formulas of ${\mathcal {L}}$, as follows. Our *c* in ${\mathfrak {F}}$ above is a function $c: {\mathcal {L}}_{AT} \rightarrow {\mathcal {C}}$, such that if $p \in {\mathcal {L}}_{AT}$, then $c(p) \in \{x \in {\mathcal {C}} | Atom(x)\}$: atomic contents are assigned to atomic formulas (this is an idealization, for grammatically simple sentences of ordinary language can be about intuitively complex contents; but it will streamline the discussion below). Next, *c* is extended to the whole of ${\mathcal {L}}$ as follows. if ${{\mathfrak {A}}}{{\mathfrak {t}}}\phi = \{p_{1},\ldots , p_{n}\}$, the set of atoms in $\phi$, then:

- $$c(\phi ) = \oplus {{\mathfrak {A}}}{{\mathfrak {t}}}\phi = c(p_{1}) \oplus \cdots \oplus c(p_{n})$$

A formula is about what its atoms, taken together, are about. This mereology of contents (of which a more refined version is Peter Hawke’s issue-based theory: see Hawke (2017)) entails, by induction on the construction of formulas, that $c(\phi ) = c(\lnot \lnot \phi )$ (recall Frege on the *Sinn*-preservation of Double Negation); but also that $c(\phi ) = c(\lnot \phi )$: a formula is about what its negation is about. And not only $c(\phi \wedge \psi ) = c(\phi \wedge \psi )$, but also, e.g., $c(\phi \wedge \psi ) = c(\phi ) \oplus c(\psi ) = c(\phi \vee \psi )$. In the literature, these are often taken as key requirements for a good account of aboutness- or content-inclusion (see Yablo 2014, p. 42; Fine 2015, p. 1).^5^

A *model*${\mathfrak {M}}$ = $\langle W, \{R_{\phi } \ | \ \phi \in {\mathcal {L}}\}, {\mathcal {C}}, \oplus , c, \Vdash \rangle$ is a frame with an interpretation $\Vdash \ \subseteq W \times {\mathcal {L}}_{AT}$, relating worlds to atoms: we read ‘$w \Vdash p$’ as meaning that *p* is true at *w*, ‘$w \,\nVdash\, p$’ as $\sim w\Vdash p$. $\Vdash$ is extended to all formulas of ${\mathcal {L}}$ via the recursive semantic clauses:

- (S$$\lnot $$) $$w \Vdash \lnot \phi \Leftrightarrow w \,\nVdash\, \phi $$
- (S$$\wedge $$) $$ w \Vdash \phi \wedge \psi \Leftrightarrow w \Vdash \phi \ \& \ w \Vdash \psi $$
- (S$$\vee $$) $$w \Vdash \phi \vee \psi \Leftrightarrow w \Vdash \phi \ or \ w \Vdash \psi $$
- (S$$\prec $$) $$w \Vdash \phi \prec \psi \Leftrightarrow \forall w_{1} (w_{1} \Vdash \phi \Rightarrow w_{1} \Vdash \psi )$$
- (S*B*) $$ w \Vdash B^{\phi }\psi \Leftrightarrow \forall w_{1} (wR_{\phi }w_{1} \Rightarrow w_{1} \Vdash \psi ) \ \& \ c(\psi ) \le c(\phi )$$

Read ‘$wR_{\phi }w_{1}$’ as meaning that $w_{1}$ is, from *w*’s viewpoint, one of the most plausible worlds where $\phi$ is true. (S*B*) can be equivalently expressed using set-selection functions (as in Lewis 1973, pp. 57–60). Each $\phi \in {\mathcal {L}}$ comes with a function $f_{\phi }: W \rightarrow {\mathcal {P}}(W)$ selecting the set of most plausible $\phi$-worlds, $f_{\phi }(w) = \{w_{1} \in W | wR_{\phi }w_{1} \}$. If $|\phi | = \{w \in W | w \Vdash \phi \}$, we can compactly rephrase the clause for *B* as:

- (S*B*) $$ w \Vdash B^{\phi }\psi \Leftrightarrow f_{\phi }(w) \subseteq |\psi | \ \& \ c(\psi ) \le c(\phi )$$

The two formulations are equivalent as $wR_{\phi }w_{1} \Leftrightarrow w_{1} \in f_{\phi }(w)$. However, either formulation is at times handier than the other to make specific points. For a conditional belief in $\psi$ given $\phi$, or a disposition to believe $\psi$ after revising by $\phi$, we ask, thus, two things at once. First, we have a truth-conditional requirement: that $\psi$ be true throughout the most plausible worlds where $\phi$ is true. Second, we have a content containment requirement: that $\psi$ allows no content alien to $\phi$ to sneak in.

For the $R_{\phi }$’s or $f_{\phi }$’s to *really* capture plausibility, or disposition to revise one’s beliefs, we characterize them via a Lewisian *system of spheres* as in Lewis (1973, pp. 57–59). In the Lewisian semantics for counterfactuals, sphere systems or, equivalently, orderings of worlds, are to capture objective world similarity. In the context of belief revision and in models inspired by the Grove (1988) reformulation, spheres are to represent, instead, subjective dispositions to revise beliefs, or preferred ‘fallback’ positions after revisions, hence our talk of plausibility above. In Leitgeb and Segerberg’s words:

In the case of belief revision the agent tries to change his beliefs in a manner such that the worlds that he subsequently believes to be in comprise the *subjectively most plausible deviation* from the worlds he originally believed to inhabit. (Leitgeb and Segerberg 2005, p. 185)

We thus have a function, $, assigning to each *w* a finite set of subsets of *W* (the spheres), $$\$(w) = \{S^{w}_{0}, S^{w}_{1},\ldots , S^{w}_{n}\}$$, with $$ n \in {\mathbb {N}}$$, such that $$S^{w}_{0} \subseteq S^{w}_{1} \subseteq \cdots \subseteq S^{w}_{n} = W$$. Next, for each $$\phi \in {\mathcal {L}}$$ and $$w \in W$$, $$f_{\phi }(w)$$ is characterized as follows: if $$|\phi | = \emptyset $$, then $$f_{\phi }(w) = \emptyset $$. Otherwise, $$f_{\phi }(w) = S^{w}_{i} \cap |\phi |$$, where $$S^{w}_{i} \in \$(w)$$ is the smallest sphere such that $$S^{w}_{i} \cap |\phi | \ne \emptyset $$. Because the spheres around each *w* are assumed to be finite, the system satisfies Lewis’ Limit Assumption: the existence of a smallest such $$S^{w}_{i}$$ for each $$w \in W$$ and $$\phi \in {\mathcal {L}}$$ is automatically guaranteed.

The system satisfies various conditions,^6^ some of which will be useful in the following:

- (C1) $$f_{\phi }(w) \subseteq |\phi |$$
- (C2) $$|\phi | \ne \emptyset \Rightarrow f_{\phi }(w) \ne \emptyset $$
- (C3) $$ f_{\phi }(w) \subseteq |\psi | \ \& \ f_{\psi }(w) \subseteq |\phi | \Rightarrow f_{\phi }(w) = f_{\psi }(w)$$
- (C4) $$f_{\phi }(w) \cap |\psi | \ne \emptyset \Rightarrow f_{\phi \wedge \psi }(w) \subseteq f_{\phi }(w)$$

Notice that we do not demand that $w \in S^{w}_{0}$, that is, the relevant world be in the innermost sphere: in Lewis’ terminology, we have a system of spheres which is not even weakly centered. That’s because our spheres do not express objective world similarity, but subjective world plausibility, or belief entrenchment. The innermost sphere at the core, $S^{w}_{0}$, gives the most plausible worlds for the agent located at *w*. *w* itself need not be among the innermost worlds, for the agent may have false beliefs.

Finally, logical consequence is defined the standard way, as truth preservation at all worlds of all models (based on our system of spheres generated by $—we will omit mentioning it each time). With $$\Sigma $$ a set of formulas:

$\Sigma \vDash \psi \Leftrightarrow$ in all models ${\mathfrak {M}}$ = $\langle W, \{R_{\phi } \ | \ \phi \in {\mathcal {L}}\}, {\mathcal {C}}, \oplus , c, \Vdash \rangle$ and for all $w \in W$: $w \Vdash \phi$ for all $\phi \in \Sigma \Rightarrow w \Vdash \psi$

For single-premise entailment, I write $\phi \vDash \psi$ for $\{\phi \} \vDash \psi$. As a special case, logical validity, $\vDash \phi$, truth at all worlds of all models, is $\emptyset \vDash \phi$, entailment by the empty set of premises.^7^

The logic induced by the semantics for the extensional operators is just classical propositional, with $\prec$ a strict S5-like conditional (i.e., one equivalent to the necessitation of a material conditional, where the relevant necessity is S5). The novelty comes with $B^{\phi }\psi$, whose logical behavior we are now going to unpack.

As we shall see, the *B* operator is essentially non-monotonic. As we go through its features, thus, it will be worth comparing^8^ the pattern of validities and invalidities we meet with the general framework for non-monotonic logics of Kraus et al. (1990). Their seminal work investigates non-monotonic consequence relations ‘$\phi \mid\!\sim \psi$’ (read: ‘$\psi$ is a plausible consequence of $\phi$’, or ‘Given $\phi$, one defeasibly jumps to the conclusion that $\psi$’). Their basic non-monotonic system, called **C**, satisfies what, according to Gabbay (1985), are three minimal inferential schemata any non-monotonic consequence must comply with, namely Reflexivity ($\phi \mid\! \sim \phi$), Cut (If $\phi \mid\! \sim \psi$ and $\phi \wedge \psi \mid\! \sim \chi$, then $\phi \mid\! \sim \chi$) and Weak or Cautious Monotonicity (If $\phi \mid\! \sim \psi$ and $\phi \mid\! \sim \chi$, then $\phi \wedge \psi \mid\! \sim \chi$). But **C** also satisfies two more conditions, called Left Logical Equivalence (If $\phi$ and $\psi$ are logically equivalent, and $\phi \mid\! \sim \chi$, then $\psi \mid\! \sim \chi$) and Right Weakening (If $\phi \mid\! \sim \psi$, and $\psi$ logically entails $\chi$, then $\phi \mid\! \sim \chi$). We will see that our hyperintensional belief operator *B* does comply with the minimal Gabbay conditions. However, the counterparts of Left Logical Equivalence and Right Weakening fail for it in our semantics—-and rightly so, for their failure is at the core of the hyperintensionality of *B*.

## Success, Conjunction, Non-monotonicity

To begin with, Condition C1 guarantees that our belief operator satisfies a principle corresponding to the Success postulate of AGM:

(Success) $$\vDash\; B^{\phi }\phi $$

[*Proof* By C1, for any *w* and $w_{1}$, $wR_{\phi }w_{1} \Rightarrow w_{1} \Vdash \phi$, and of course $c(\phi ) \le c(\phi )$.]

Success guarantees that, after revising by $\phi$, one does believe $\phi$. The next validities show that conditional belief is closed with respect to conjunction introduction and elimination:

(Simplification) $$B^{\phi }(\psi \wedge \chi ) \,\vDash\, B^{\phi }\;\psi $$ $$B^{\phi }(\psi \wedge \chi ) \,\vDash\, B^{\phi }\chi $$

[*Proof* we do the first one (for the second, replace $\psi$ with $\chi$ appropriately). Let $w \Vdash B^{\phi }(\psi \wedge \chi )$. By (S*B*), for all $w_{1}$ such that $wR_{\phi }w_{1}$, $w_{1} \Vdash \psi \wedge \chi$, thus by (S$\wedge$), $w_{1} \Vdash \psi$. Also, $c(\psi \wedge \chi ) = c(\psi ) \oplus c(\chi ) \le c(\phi )$, thus $c(\psi ) \le c(\phi )$. Then, by (S*B*) again, $w \Vdash B^{\phi }\psi$.]

If, after revising by $\phi$, you believe $\psi$ and $\chi$, then you believe $\psi$ (and you also believe $\chi$) after the same revision. The companion of Simplification is:

(Adjunction) $$\{B^{\phi }\psi , B^{\phi }\chi \} \vDash B^{\phi }(\psi \wedge \chi )$$

[*Proof* let $w \Vdash B^{\phi }\psi$ and $w \Vdash B^{\phi }\chi$, that is, by (S*B*): for all $w_{1}$ such that $wR_{\phi }w_{1}$, $w_{1} \Vdash \psi$ and $w_{1} \Vdash \chi$, so by (S$\wedge$) $w_{1} \Vdash \phi \wedge \psi$. Also, $c(\psi ) \le c(\phi )$ and $c(\chi ) \le c(\phi )$, thus $c(\psi ) \oplus c(\chi ) = c(\psi \wedge \chi ) \le c(\phi )$. Then, by (S*B*) again, $w \Vdash B^{\phi }(\psi \wedge \chi )$.]

If, after revising by $\phi$, you believe $\psi$ and after revising by $\phi$, you believe $\chi$, then you believe $\psi$ and $\chi$ together after the same revision. Simplification and Adjunction mirror in the language the idea that, when one comes to believe a certain content, one believes all of its parts and vice versa. This does impose an amount of *computational* idealization. There may be a computational or broadly syntactic difference between believing that $\phi$ and that $\psi$ separately, and believing them together, and even between believing them together in the order $\phi \wedge \psi$ and in the order $\psi \wedge \phi$. For instance, ‘a computer program that can determine whether $\phi \wedge \psi$ follows from some initial premises in time $\tau$ might not be able to determine whether $\psi \wedge \phi$ follows from those premises in time $\tau$’ (Fagin and Halpern 1988, p. 53, notation modified).

However, this is no difference concerning the *scenario* represented in the mind of an intentional agent. Modulo the different attitudes one can have towards the same content, representing in the mind a configuration of objects and properties making $\phi \wedge \psi$ true is the same as representing one making $\psi \wedge \phi$ true. Levesque (1984)’s classic criticism of merely syntactic approaches to knowledge and belief hinges on this. He makes the point with respect to disjunction:

The syntactic approach [...] is too *fine-grained* in that it considers any two sets of sentences as distinct semantic entities and, consequently, different belief sets. Consider, for example, the disjunction of $\phi$ and $\psi$. There is no reason to suppose that $B(\phi \vee \psi ) \equiv B(\psi \vee \phi )$ would be *valid* given a syntactic understanding of *B* since $\phi \vee \psi$ may be in the belief set while $\psi \vee \phi$ may not. [But] if we consider intuitively what ‘*It is believed that either*$\phi$*or*$\psi$*is true*.’ is saying, the order seems to be completely irrelevant [...] $\phi \vee \psi$ is believed iff $\psi \vee \phi$ is because these are two lexical notations for the *same* belief. (Levesque 1984, pp. 199–201, notation modified)

If believing has to be taken, not just as mental symbol manipulation, but as an intentional state endowed with aboutness, then when one believes that $\psi \wedge \chi$ one intends a certain scenario in which both the situation described by $\psi$ and the situation described by $\chi$ obtain (and one takes the actual world to be like that). Vice versa, when one believes that $\psi$ and that $\chi$, one intends a certain scenario, one in which $\psi$ obtains and $\chi$ obtains as well (and one takes, etc.). Then the scenario will be such that it includes both contents, and one will believe them together. This provides intuitive justification for Simplification and Adjunction.

Another conjunction-involving feature (this time, an invalidity) displays the non-monotonic features of belief revision. Our operator is non-monotonic in this sense:

$$\begin{aligned} B^{\phi }\psi \,\nvDash\, B^{\phi \wedge \chi }\psi \end{aligned}$$

[*Countermodel* let $W = \{w, w_{1}\}$, *w*$R_{p}$-accesses nothing, $wR_{p \wedge r}w_{1}$, $w_{1} \,\nVdash\, q$, $c(p) = c(q) = c(r)$. Then $w \Vdash B^{p}q$, but $w \,\nVdash\, B^{p \wedge r}q$.]

Content is preserved here, for in general if $c(\psi ) \le c(\phi )$, then also $c(\psi ) \le c(\phi ) \oplus c(\chi ) = c(\phi \wedge \chi )$. What does the trick is the variability in strictness of our operator: $f_{\phi }(w)$ need not be the same as $f_{\phi \wedge \chi }(w)$. After revising your beliefs via the new information that Mike is in Strasbourg, you come to believe that he is in France. But if you are informed that Mike is in Strasbourg *and* that the city has been annexed by Germany, you will not come to believe that he is in France.

## Disjunction and Indeterminacy

When, after revising by $\phi$, one comes to believe that $\psi$, one does not thereby believe a disjunction between the latter and an unrelated $\chi$, although any formula logically entails the disjunction between itself and something else. Intuitively enough, the believer need not be aware of that disconnected $\chi$ at all, or that $\chi$ might be irrelevant to the agent’s belief revision policy. Thus we need, and we get:

$$\begin{aligned} B^{\phi }\psi \,\nvDash\, B^{\phi }(\psi \vee \chi ). \end{aligned}$$

[*Countermodel* let $W = \{w, w_{1}\}$, $wR_{p}w_{1}$, $w_{1} \Vdash q$, $c(p) = c(q) \ne c(r)$. Then $c(q) \le c(p)$, so by (S*B*), $w \Vdash B^{p}q$. But $c(q \vee r) = c(q) \oplus c(r) \nleq c(p)$, thus $w \,\nVdash\, B^{p}(q \vee r)$.]

Notice that the inference fails for the right reason: although $\psi \vDash \psi \vee \chi$, disjunction brings in irrelevant, alien content. When informed that Mike is not in France, as you thought, but in New Zealand, you come to believe that Mike is in Oceania. You do not thereby automatically believe that Mike is either in Oceania or in planet Kepler-442b (you may never have heard of Kepler-442b to begin with), though the former entails the latter.

Another disjunction-involving issue has to do with the fact that beliefs can be ‘nonprime’ due to indeterminacy in the information via which we revise. After being informed that Mike landed in New Zealand, you come to believe that he is either in the North Island or in the South Island, but you are not sure which one it is. Thus, you don’t believe him to be in either rather than the other. So we need, and we get:

$$\begin{aligned} B^{\phi }(\psi \vee \chi ) \,\nvDash\, B^{\phi }\psi \vee B^{\phi }\chi \end{aligned}$$

[*Countermodel* let $W = \{w, w_{1}, w_{2}\}$, $wR_{p}w_{1}$, $wR_{p}w_{2}$, $w_{1} \Vdash q$ but $w_{1} \,\nVdash\, r$, $w_{2} \Vdash r$ but $w_{2} \,\nVdash\, q$, $c(p) = c(q) = c(r)$. Then by (S$\vee$), $w_{1} \Vdash q \vee r$ and $w_{2} \Vdash q \vee r$, so for all $w_{x}$ such that $wR_{p}w_{x}$, $w_{x} \Vdash q \vee r$. Also, $c(q \vee r) = c(q) \oplus c(r) \le c(p)$, thus by (S*B*), $w \Vdash B^{p}(q \vee r)$. However, $w \,\nVdash\, B^{p}q$ and $w \,\nVdash\, B^{p}r$ for both *q* and *r* fail at some $R_{p}$-accessible world. Thus by (S$\vee$), $w \,\nVdash\, B^{p}q \vee B^{p}r$.]

Here, too, the inference fails for the right reason. Content-inclusion works: when one believes that either $\psi$ or $\chi$, one’s state of the mind is about both things, just as when one believes that $\psi$ and $\chi$ ($c(\psi \wedge \chi ) = c(\psi ) \oplus c(\chi ) = c(\psi \vee \chi )$). But the different worlds one has access to will fill in the unspecified details in different ways. There can be a world where $\psi$ but not $\chi$, and a world where $\chi$ but not $\psi$, and both can be among the most plausible $\phi$-worlds.

## Hyperintensionality

I now come to interesting invalidities highlighting the hyperintensional features of belief revision in our models. To begin with, one’s coming to believe that $\psi$ given $\phi$ is not entailed by the corresponding strict conditional or implication:

$$\begin{aligned} \phi \prec \psi \,\nvDash\, B^{\phi }\psi \end{aligned}$$

[*Countermodel* let $W = \{w, w_{1}\}$, $wR_{p}w_{1}$, $w \,\nVdash\, p$, $w_{1} \Vdash q$, $c(p) \ne c(q)$. By Condition C1, $w_{1} \Vdash p$. Now $|p| \subseteq |q|$, thus by (S$\prec$), $w \Vdash p \prec q$. But although $f_{p}(w) \subseteq |q|$, $c(q) \nleq c(p)$, thus $w \,\nVdash\, B^{p}q$.]

The strict conditional is ‘irrelevant’, in the sense criticized in Anderson and Belnap (1975), Anderson et al. (1992)’s relevant logic programme: even when all the $\phi$-worlds are $\psi$-worlds (thus, all the most plausible $\phi$-worlds are $\psi$-worlds), $\psi$ may have little to do with $\phi$. Thus, one can revise one’s beliefs by $\phi$ but fail to come to believe that $\psi$, although there is no way for $\phi$ to be true while $\psi$ is not.

For similar reasons, what is strictly implied by what we come to believe after revision is not perforce believed in its turn—thus, the counterpart of Kraus et al. (1990)’s Right Weakening fails:

$$\begin{aligned} \{B^{\phi }\psi , \psi \prec \chi \} \,\nvDash\, B^{\phi }\chi \end{aligned}$$

[*Countermodel* let $W = \{w, w_{1}\}$, $wR_{p}w_{1}$, $w \,\nVdash\, q$, $w_{1} \Vdash q$, $w_{1} \Vdash r$, $c(p) = c(q) \ne c(r)$. Then $f_{p}(w) \subseteq |q|$ and $c(q) \le c(p)$, thus by (S*B*), $w \Vdash B^{p}q$. Also, $|q| \subseteq |r|$, thus by (S$\prec$), $w \Vdash q \prec r$. But although $f_{p}(w) \subseteq |r|$, $c(r) \nleq c(p)$, thus $w \,\nVdash\, B^{p}r$.]

Here, too, the failure happens for the right reason: although all the $\psi$-worlds are $\chi$-worlds, thus all the most plausible $\phi$-worlds which are $\psi$-worlds are $\chi$-worlds, the strict conditional can change the subject. Thus after revision by $\phi$ one can believe $\psi$ but fail to believe $\chi$, although there is no way for $\psi$ to be true while $\chi$ is not. After spotting a furry animal running in your garden, you come to believe that there’s a woodchuck behind home. You need not come to believe, too, that there’s a groundhog behind home, although there’s no way for something to be a woodchuck without it being a groundhog: you are just unaware of this latter fact.

Things are different if, after revision, one comes to believe the strict implication *itself*. This principle, which might be called Closure under Believed Implication, is valid:

(CBI) $$\{B^{\phi }\psi , B^{\phi }(\psi \prec \chi )\} \vDash B^{\phi }\chi $$

[*Proof* let $w \Vdash B^{\phi }\psi$ and $w \Vdash B^{\phi }(\psi \prec \chi )$. By the former and (S*B*), for all $w_{1}$ such that $wR_{\phi }w_{1}$, $w_{1} \Vdash \psi$, and $c(\psi ) \le c(\phi )$. By the latter and (S*B*) again, for all $w_{1}$ such that $wR_{\phi }w_{1}$, $w_{1} \Vdash \psi \prec \chi$, thus given (S$\prec$) for all $w \in W$, if $w \Vdash \psi$ then $w \Vdash \chi$. So in particular for all $w_{1}$ such that $wR_{\phi }w_{1}$, $w_{1} \Vdash \chi$. Also, $c(\psi \prec \chi ) = c(\psi ) \oplus c(\chi ) \le c(\phi )$, thus $c(\chi ) \le c(\phi )$. Thus by (S*B*), $w \Vdash B^{\phi }\chi$.]

Here both $\psi$ and $\psi \prec \chi$ come to be believed: after revision by $\phi$, the agent believes both that $\psi$ and that there is no way for $\psi$ to be true while $\chi$ is not. Thus, the agent’s intentional state is about $\psi$ as well as $\chi$. Then the agent also comes to believe that $\chi$, *under the same revision by*$\phi$ (the final proviso is essential: given the non-monotonic features of *B* highlighted above, the inference would not be valid anymore if the revision input were allowed to change across the involved formulas). As remarked by Yablo (2014, p. 117) (who makes the point about knowledge), this is plausible enough: coming to believe something entails coming to believe the believed implications that do not change the subject.

Next, also the counterpart of Kraus et al. (1990)’s Left Logical Equivalence fails (where $\phi \equiv \psi$ abbreviates $\phi \prec \psi \wedge \psi \prec \phi$):

$$\begin{aligned} \{B^{\phi }\chi , \phi \equiv \psi \} \,\nvDash\, B^{\psi }\chi \end{aligned}$$

[*Countermodel* let $W =\{w, w_{1}\}$, $wR_{p}w_{1}$, $wR_{q}w_{1}$, $w \,\nVdash\, p$, $w \,\nVdash\, q$, $w_{1}\Vdash r$, $c(p) = c(r) \ne c(q)$. Then $f_{p}(w) \subseteq |r|$ and $c(r) \le c(p)$, thus by (S*B*), $w \Vdash B^{p}r$. Also, by Condition C1, $w_{1} \Vdash p$ and $w_{1} \Vdash q$, thus $|p| \subseteq |q|$ and $|q| \subseteq |p|$. Then by (S$\prec$) and (S$\wedge$), $w \Vdash p \equiv q$. But although $f_{q}(w) \subseteq |r|$, $c(r) \nleq c(q)$, thus $w \,\nVdash\, B^{q}r$.]

Again, failure of aboutness does the trick of differentiating necessarily equivalent contents. This invalidity allows a proper appreciation of framing effects in our semantics: after being informed that one’s probability of making it to the short list is 1 / 3, one believes that one should apply for the job. But, against such principles as AGM’s (${{\hbox {K}}}*6$), after being informed that one’s probability of failing the short list is 2 / 3, one does not believe that it’s worth applying. There is no way that the chances of making it are 1 / 3 without the chances of failing being 2 / 3 and vice versa, but one has been caught into a framing effect.

## Revising by Inconsistent Information

In our framework, belief revision is not automatically trivialized by incoming inconsistent information. We already know that, via its content-preservation constraints, our belief revision has an element of relevance in the sense of relevant logics. Our S5-ish strict implication is ‘explosive’, $\nvDash (\phi \wedge \lnot \phi ) \prec \psi$ (trivially: for all *w*, $w\,\nVdash\, \phi \wedge \lnot \phi$.) But, against principles such as AGM’s (${{\hbox {K}}}*5$), the following ensures that we do not come to believe arbitrary, irrelevant things just because we have taken on board explicitly inconsistent information:

$$\begin{aligned} \,\vDash\, B^{\phi \wedge \lnot \phi }\psi \end{aligned}$$

[*Countermodel* let $W = \{w\}$, $c(p) \ne c(q)$. $|p \wedge \lnot p| = \emptyset$, thus $f_{p \wedge \lnot p}(w) = \emptyset \le |q|$. However, $c(q) \nleq c(p \wedge \lnot p) = c(p) \oplus c(\lnot p) = c(p)$. Thus, by (S*B*), $w \,\nVdash\, B^{p \wedge \lnot p}q$.]

Although there is no possible world where a contradiction is true, inconsistent information may still be about *something*. In general $\phi \wedge \lnot \phi$ is not contentless: its content is whatever $\phi$ is about, and this may not include the content of $\psi$ (*Snow is white and not white* is about snow’s being white, not about grass’ being purple).^9^

For similar reasons, when we revise by $\phi$ we do not automatically come to believe logical validities whose content is irrelevant with respect to $\phi$, e.g.:

$$\begin{aligned}&\,\nvDash\, B^{\phi }(\psi \prec \psi )\\&\,\nvDash\, B^{\phi }(\psi \vee \lnot \psi ) \end{aligned}$$

[*Countermodel* (we do the former, the latter is analogue): let $W = \{w\}, c(p) \ne c(q)$. Then although (trivially) $f_{p}(w) \subseteq |q \prec q|$, $c(q \prec q) = c(q) \oplus c(q) = c(q) \nleq c(p)$. Thus by (S*B*), $w \,\nVdash\, B^{p}(q \prec q)$.]

## Equivalents in Plausibility, Cut, Cautious Monotonicity

I close with some important validities warranted with the help of Condition C3 above.^10^ One may be called a Principle of Equivalents in Plausibility. It allows a limited recovery of the idea encoded in principles like AGM’s (${{\hbox {K}}}*6$):

(PEP) $$\{B^{\phi }\psi , B^{\psi }\phi , B^{\phi }\chi \} \vDash B^{\psi }\chi $$

[*Proof* suppose $w \,\Vdash\, B^{\phi }\psi$, $w \,\Vdash\, B^{\psi }\phi$, $w \Vdash B^{\phi }\chi$. By (S*B*), these entail, respectively, (a) $f_{\phi }(w) \subseteq |\psi |$ and $c(\psi ) \le c(\phi )$, (b) $f_{\psi }(w) \subseteq |\phi |$ and $c(\phi ) \le c(\psi )$, (c) $f_{\phi }(w) \subseteq |\chi |$ and $c(\chi ) \le c(\phi )$. From (a) and (b) we get $f_{\phi }(w) = f_{\psi }(w)$ (by Condition C3) and $c(\phi ) = c(\psi )$ (by antisymmetry of content parthood). From these and (c) we get $f_{\psi }(w) \subseteq |\chi |$ and $c(\chi ) \le c(\psi )$. Thus by (S*B*) again, $w \Vdash B^{\psi }\chi$.]

‘Equivalents in plausibility’ are formulas $\phi$ and $\psi$ such that, when we revise by either, we come to believe the other. PEP says that such equivalents can be replaced *salva veritate* as inputs for belief revision: when we revise by either, we come to have the same beliefs. This seems plausible. $B^{\phi }\psi$: informed that Mike is unmarried, you come to believe that he is a bachelor. Also, $B^{\psi }\phi$: informed that Mike is a bachelor, you come to believe that he is unmarried. Suppose $B^{\phi }\chi$: informed that Mike is unmarried, you come to believe that he has no marriage allowance. Then the same happens if you are informed that he is a bachelor, $B^{\psi }\chi$.

While general transitivity fails for our *B* as a consequence of its variable strictness, C3 validates a kind of limited transitivity or Cut principle for belief revision:

(CUT) $$\{ B^{\phi }\psi , B^{\phi \wedge \psi }\chi \} \vDash B^{\phi }\chi $$

[*Proof* suppose (a) $w \Vdash B^{\phi }\psi$ and (b) $w \Vdash B^{\phi \wedge \psi }\chi$. From (a), Success, and Adjunction we get $w \Vdash B^{\phi }(\phi \wedge \psi )$, thus, by (S*B*), $f_{\phi }(w) \subseteq |\phi \wedge \psi |$ and $c(\phi \wedge \psi ) \le c(\phi )$. Also, $w \Vdash B^{\phi \wedge \psi }\phi$ (from Success $\vDash B^{\phi \wedge \psi }(\phi \wedge \psi )$ and Simplification). By (S*B*) again,$f_{\phi \wedge \psi }(w) \subseteq |\phi |$ and (of course) $c(\phi ) \le c(\phi \wedge \psi )$. Thus, by Condition C3 $f_{\phi }(w) = f_{\phi \wedge \psi }(w)$, and $c(\phi \wedge \psi ) = c(\phi )$ (by antisymmetry of content parthood). Next, from (b) and (S*B*) again, $f_{\phi \wedge \psi }(w) \subseteq |\chi |$ and $c(\chi ) \le c(\phi \wedge \psi )$. Therefore, $f_{\phi \wedge \psi }(w) = f_{\phi }(w) \subseteq |\chi |$ and $c(\chi ) \le c(\phi ) = c(\phi \wedge \psi )$. Thus by (S*B*) again, $w \Vdash B^{\phi }\chi$.]

C3 also validates a converse principle of Cautious Monotonicity:

(CM) $$\{B^{\phi }\psi , B^{\phi }\chi \} \,\vDash\, B^{\phi \wedge \psi }\chi $$

[*Proof* suppose (a) $w \Vdash B^{\phi }\psi$ and (b) $w \Vdash B^{\phi }\chi$. From (a), Success ($\vDash B^{\phi }\phi$), and Adjunction, we get $w \Vdash B^{\phi }(\phi \wedge \psi )$, thus by (S*B*), $f_{\phi }(w) \subseteq |\phi \wedge \psi |$. Also, $w \Vdash B^{\phi \wedge \psi }\phi$ (from Success $\vDash B^{\phi \wedge \psi }(\phi \wedge \psi )$ and Simplification), so by (S*B*) again, $f_{\phi \wedge \psi }(w) \subseteq |\phi |$. Then, by Condition C3, $f_{\phi }(w) = f_{\phi \wedge \psi }(w)$. From (b) and (S*B*) again, we get $f_{\phi }(w) \subseteq |\chi |$, thus $f_{\phi \wedge \psi }(w) \subseteq |\chi |$. Also, $c(\chi ) \le c(\phi ) \oplus c(\psi ) = c(\phi \wedge \psi )$. Thus, $w \Vdash B^{\phi \wedge \psi }\chi$.]

Cut and Cautious Monotonicity are defended in Board (2004, p. 56), as ‘principles of informational economy’. They correspond to the Cut and Cautious Monotonicity principles for non-monotonic logic considered in Kraus et al. (1990). By satisfying these as well as Reflexivity (that’s our Success principle above), our $B^{\phi }\psi$ complies, thus, with Gabbay (1985)’s minimal conditions for non-monotonic entailments.

## Conclusion and Further Work

This work was just an initial exploration of a simple formal set-up for hyperintensional belief revision that looks, in my view, promising: it preserves some good features of more standard approaches while overcoming some problems due to agent idealization, and achieves so by sticking to a fairly classical modal framework. The first obvious direction of further work would consist in coming up with a proof system sound and complete with respect to the above semantics, or variations thereof.

A second direction would be to develop the set-up following the lines pursued in dynamic doxastic and epistemic logics such as Baltag and Solecki (1998), Leitgeb and Segerberg (2005), Baltag and Smets (2008), etc. These may include: having a multiplicity of agents; modeling different kinds of belief revision (irrevocable, minimal, etc.: see e.g. van Ditmarsch 2005); and above all, defining dynamic operators whose semantics is given via model transformations, but which include content-containment requirements like those of our semantics above, allowing many hyperintensional distinctions invisible in ‘merely modal’ models.^11^

A third direction^12^ would be to go first-order. In the current propositional set-up, contents can be taken as situations, and fusions thereof. In a semantics for a predicative language, contents could be thought of in terms of objects (what singular terms are about) and concepts (what predicates are about), and fusions thereof. In the hyperintensional camp, of course, the main topic would be the one of Frege’s informative identities: how Lois can (come to) believe that Clark Kent is in love with her, without (coming to) believe that Superman is in love with her.

## Footnotes

1 I have presented versions of the semantics used in this paper at the European Conference of Analytic Philosophy in Munich; at the workshop ‘Doxastic Agency and Epistemic Logic’ in Bochum; at the conference ‘Conceivability and Modality’ at the University of Rome-La Sapienza; at the workshop ‘Imagination and Mental Imagery in Epistemology’ at the University of Antwerp; at the Seminar of the Arche Logic Group, University of St Andrews; at the Tokyo Forum for Analytic Philosophy, University of Tokyo; at the Center for Logic Language and Cognition, University of Turin; and at the Logic of Conceivability Seminar at the University of Amsterdam. I am very grateful to all those who provided comments, including two anonymous referees of this journal. Special thanks to the Logic of Conceivability gang, in particular to Chris Badura, Ilaria Canavotto, Jorge Ferreira, Peter Hawke, Karolina Krzyzanowska, Martin Lipman, Aybüke Ozgün, Tom Schoonen, Anthi Solaki.

2 If, as claimed e.g. by Floridi (2005), information is factive, that is, being informed that $\phi$ entails $\phi$, then there cannot be inconsistent information unless, as believed by dialetheists like Priest (1987), there are true contradictions. In the literature on belief revision, however, a weaker sense of information is often adopted, whereby (declarative) information is meaningful data, not perforce truthful. This is connected to what is sometimes called ‘soft information’, e.g., in van Benthem (2011), van Benthem and Smets (2015).

3 A retort one hears at times, is that *B* in (H) expresses not knowledge or belief, but rather what an agent is logically committed to, given what it knows or believes. A similar answer is found in belief revision research, e.g., in Levi (1991). This leaves us in want of a logical account of *knowledge* and *belief* for real agents, as opposed to some conditional commitment.

4 I do not aim at modeling agents free from any kind of logical omniscience, so our theory still includes an amount of idealization. It seems to me that a framework for epistemic logic should strike a balance between two desiderata that pull in opposite directions. On the one hand, it should yield enough logical structure to justify the use of formal logical tools. On the other hand, it should allow us to reason about the epistemic states of agents that are of genuine interest. The former requirement pulls towards modeling infallible agents with heavily idealized cognitive powers. The latter can easily lead to very complex frameworks, or to logics that are too weak to be of serious interest.

5 As highlighted by an anonymous referee of this journal, there are connections between such a modeling of aboutness via a mereology of contents, and the formalization of *awareness* in awareness logics (the seminal work in this area is Fagin and Halpern 1988). In a sense, this is not surprising, given the intuitive link between the idea of concepts an epistemic agent is aware of, and contents its intentional states are about. The version of awareness logic closest to our framework is the one in terms of ‘awareness generated by primitive propositions’ (see Schipper 2015, p. 81), where an agent is aware of a formula $\phi$ just in case it is aware of all of its atomic constituents, taken together. The essential role of content-inclusions will be to allow us to capture a certain *relevance* in belief revision. Works formalizing how belief change is sensitive to issues of relevance include Del Cerro and Herzig (1996), Chopra and Parikh (2000), Hansson and Wassermann (2002), Peppas et al. (2015).

6 *Proof* by checking Priest (2001), pp. 91, 92.

7 I have used a semantics quite similar to the one just presented (save for the lack of a plausibility metric on worlds) in Berto (2017a), to model a sort of imagination, understood as reality-oriented mental simulation. An antecedent of this kind of semantics, based on the idea of content-preservation, is to be found in works on `analytic implication' such as Angell (1977), Fine (1986), French (2017). For a useful survey, see Ferguson (2014).

8 As rightly suggested by an anonymous referee of this journal.

9 Here’s where non-classical frameworks get a revenge, though. Even if our *B* is technically not explosive, it does satisfy ‘small explosion’ principles like $\vDash B^{\phi \wedge \lnot \phi \wedge \psi } \lnot \psi$ (for, trivially, $\phi \wedge \lnot \phi \wedge \psi$ is true nowhere, *and* content is preserved here). A framework expanded to include non-normal or impossible worlds where a contradiction can be true would help against such small detonations. A framework expanded to include non-normal or impossible worlds where a contradiction can be true would help against such small detonations. I have used such a framework to model intentional operators in Berto (2014, 2017b).

10 The behavior in our framework of (counterparts of) AGM principles other than (${{\hbox {K}}}*1$), (${{\hbox {K}}}*2$), (${{\hbox {K}}}*5$) and (${{\hbox {K}}}*6$) is somewhat less interesting, for that’s not where the original features of the theory emerge. We will, however, mention a peculiar asymmetry related to the AGM principles (${{\hbox {K}}}*7$) and (${{\hbox {K}}}*8$). A natural counterpart of (${{\hbox {K}}}*7$) (see Board 2004, p. 55) fails in our semantics:

$$\begin{aligned} \{\lnot B^{\phi }\lnot \psi , B^{\phi \wedge \psi }\chi \} \,\nvDash\, B^{\phi }(\psi \prec \chi ) \end{aligned}$$

[*Countermodel* let $W = \{w\}, f_{p}(w) = \emptyset , f_{p \wedge q} = \emptyset , c(p) \ne c(q) = c(r)$. Then by (S*B*), $w \,\nVdash\, B^{p} \lnot q$ because $c (\lnot q) = c(q) \nleq c(p)$, so $w \Vdash \lnot B^{p} \lnot q$; and $w \Vdash B^{p \wedge q}r$, because (trivially) $f_{p \wedge q}(w) \subseteq |r|$, and $c(r) = c(q) \le c(p) \oplus c(q) = c(p \wedge q)$. However, $w \,\nVdash\, B^{p}(q \prec r)$, because $c(q \prec r) = c(q) \oplus c(r) = c(q) \nleq c(p)$.] On the other hand, a natural counterpart of (${{\hbox {K}}}*8$) (see Board 2004, Ibid), obtained by flipping premise and conclusion in the former, holds:

$$\begin{aligned} \{\lnot B^{\phi }\lnot \psi , B^{\phi }(\psi \prec \chi )\} \,\vDash\, B^{\phi \wedge \psi }\chi \end{aligned}$$

[*Proof* suppose (a) $w \Vdash \lnot B^{\phi } \lnot \psi$ and (b) $w \Vdash B^{\phi }(\psi \prec \chi )$. By (a) and (S$\lnot$), $w \,\nVdash\, B^{\phi }\lnot \psi$, that is: either $f_{\phi }(w) \nsubseteq |\lnot \psi |$, that is, $f_{\phi }(w) \cap |\psi | \ne \emptyset$, or $c(\lnot \psi ) = c(\psi ) \nleq c(\phi )$. But it can’t be the latter, because by (b) and (S*B*), $c(\psi \prec \chi ) = c(\psi ) \oplus c(\chi ) \le c(\phi )$, thus in particular $c(\psi ) \le c(\phi )$; so it must be the former. Applying Condition C5 to it, $f_{\phi \wedge \psi }(w) \subseteq f_{\phi }(w)$. By Condition C1, $f_{\phi \wedge \psi }(w) \subseteq |\phi \wedge \psi |$, so by (S$\wedge$), $f_{\phi \wedge \psi }(w) \subseteq | \psi |$. By (b) and (S*B*) again, $f_{\phi }(w) \subseteq |\psi \prec \chi |$. Putting things together: $f_{\phi \wedge \psi }(w) \subseteq f_{\phi }(w) \subseteq |\psi \prec \chi |$, so $f_{\phi \wedge \psi }(w) \subseteq |\psi \prec \chi |$; and since $f_{\phi \wedge \psi }(w) \subseteq | \psi |$, then by modus ponens $f_{\phi \wedge \psi }(w) \subseteq | \chi |$. Also, by (b) again, $c(\psi ) \oplus c(\chi ) \le c(\phi ) \le c(\phi \wedge \psi )$, thus $c(\chi ) \le c(\phi \wedge \psi )$. Thus, by (S*B*), $w \Vdash B^{\phi \wedge \psi } \chi$.]

11 In particular, as highlighted by one anonymous referee of this journal, the static semantics we currently have does not track how the content of $\psi$ might connect not only with that of $\phi$ in $B^{\phi }\psi$, but also with that of previous beliefs; and it might be that going dynamic can provide a way to model this further connection.

12 Suggested, again, by an anonymous referee of this journal.
